# Evaluating Machine Learning-Based Soft Sensors for Effluent Quality Prediction in Wastewater Treatment Under Variable Weather Conditions

**DOI:** 10.3390/s25061692

**Published:** 2025-03-08

**Authors:** Daniel Voipan, Andreea Elena Voipan, Marian Barbu

**Affiliations:** 1 Department of Computer Science and Information Technology, ‘Dunarea de Jos’ University of Galati, 800008 Galati, Romania; 2 Department of Automation, ‘Dunarea de Jos’ University of Galati, 800008 Galati, Romania; andreea.voipan@ugal.ro (A.E.V.); marian.barbu@ugal.ro (M.B.)

**Keywords:** soft sensors, artificial intelligence, wastewater treatment, effluent quality, GRU, transformer, LSTM, RF classification, weather conditions, BSM2

## Abstract

Maintaining effluent quality in wastewater treatment plants (WWTPs) comes with significant challenges under variable weather conditions, where sudden changes in flow rate and increased pollutant loads can affect treatment performance. Traditional physical sensors became both expensive and susceptible to failure under extreme conditions. In this study, we evaluate the performance of soft sensors based on artificial intelligence (AI) to predict the components underlying the calculation of the effluent quality index (EQI). We thus focus our study on three ML models: Long Short-Term Memory (LSTM), Gated Recurrent Unit (GRU) and Transformer. Using the Benchmark Simulation Model no. 2 (BSM2) as the WWTP, we were able to obtain datasets for training the ML models and to evaluate their performance in dry weather scenarios, rainy episodes, and storm events. To improve the classification of networks according to the type of weather, we developed a Random Forest (RF)-based meta-classifier. The results indicate that for dry weather conditions the Transformer network achieved the best performance, while for rain episodes and storm scenarios the GRU was able to capture sudden variations with the highest accuracy. LSTM performed normally in stable conditions but struggled with rapid fluctuations. These results support the decision to integrate AI-based predictive models in WWTPs, highlighting the top performances of both a recurrent network (GRU) and a feed-forward network (Transformer) in obtaining effluent quality predictions under different weather conditions.

## 1. Introduction

One of the main missions of environmental protection is to monitor the quality of wastewater, as both climate change and urban expansion are putting serious pressure on sewage infrastructure [1,2]. Achieving high-quality effluent, raised to national and international standards, requires a lot of effort and technology [3,4], such as continuous monitoring [5], accurate prediction tools [6], and advanced control strategies [7].

Traditional control methods available today rely on physical sensors placed at various key points in the wastewater treatment plant (WWTP) [2], ready to collect data on numerous parameters, such as dissolved oxygen, turbidity, or nitrogen compounds [8]. Although their performance has proven to be high over time, some disadvantages have emerged, such as high costs, possible mechanical disturbances, and long-term reliability in environments with extreme variations [9].

In support of these methods, recent research has been directed toward software sensors, designed to complement or even partially replace these systems [8]. Developed to integrate artificial intelligence (AI) and machine learning (ML) methods in estimating more than the effluent parameters, they promise available measurements online, real-time estimation of variables, a reduction in costs, and low risk of instrumentation and maintenance [5]. However, even the accuracy and robustness of soft sensors can be influenced by weather conditions such as rain and storms if the ML methods are not well chosen and trained [10]. Moreover, as in the case of physical sensors, large variations in WWTP flow and the amount of pollutants could influence the predictions.

Therefore, in this paper we propose to evaluate the performance of three ML models, such as Long Short-Term Memory (LSTM), Gated Recurrent Unit (GRU), and Transformer, in predicting different influent and effluent compounds involved in calculating the effluent quality index (EQI). Thus, we propose the most appropriate ML prediction method that soft sensors should use in real-life scenarios such as dry weather, rain events, and storm events. The software platform used to obtain the training data and implement the neural networks (NNs) was Benchmark Simulation Model no. 2 (BSM2) [11], an international standard model in MATLAB R2021b and Simulink version R2021b, used to simulate real operating conditions of WWTPs. In addition to the previously mentioned NNs, we integrate in BSM2 a Random Forest (RF)-based decision meta-classifier, which chooses the NN that meets the prediction performance, based on the imposed variables and the meteorological conditions. In this context, the proposed application provides a complete perspective on the capabilities that AI can use in the wastewater field.

The final goal is to provide technicians and professionals with a tool for monitoring and early prediction of the effluent that, if not properly controlled during certain weather events, can subsequently affect the supply of the population.

## 2. Materials and Methods

### 2.1. Effluent Quality Index (EQI)

One widely used method to evaluate the performance of a WWTP is the EQI, which is a numerical measure of several process parameters that, when integrated into a single overall metric, allows for effective monitoring of system operations, supports evaluations of different control strategies, and simplifies communication to the public and external stakeholders [9,12].

In BSM2, the EQI calculation is performed over a one-year dynamic simulation under different operational scenarios, after already reaching one year above a quasi-steady state and tuning the control algorithms [11]. The distinctive feature of BSM2 is the final form of the total effluent required for the EQI calculation, consisting of three separate effluent streams, each with its own flow rate and pollutant loads [13]. The main parameters on which the EQI is calculated may slightly vary depending on regional standards and research objectives, but in BSM2, the following components are taken into account—total suspended solids (TSSs), chemical oxygen demand (COD), Kjeldahl nitrogen (SNKj), nitrate nitrogen (SNO), and five-day biochemical oxygen demand (BOD5), with different weighting factors assigned to each of the three effluent streams in the BOD5 calculation [11].

As mentioned above, one thing to consider is the weighting factor assigned to each parameter, to indicate its impact on water quality. In BSM2, typical weighting values, detailed in studies such as [14], have the following values:

B_TSS_ = 2, B_COD_ = 1, B_NKj_ = 30, B_NO_ = 10, B_BOD5_ = 2. (1)

The general formula of the EQI is obtained by integrating the total effluent and the weighed pollutant concentrations, over the observation period, in the form of

(2) $$EQI=\frac{1}{t_{obs}\cdot 1000}\int_{t=245\;days}^{t=609\;days}\left(\begin{array}{c} B_{TSS}\;\cdot \;{TSS}_{e}\left(t\right)+B_{COD}\;\cdot \;{COD}_{e}\left(t\right)+B_{NKj}\;\cdot \;S_{NKj,e}\left(t\right)\\ +\;B_{NO}\;\cdot \;S_{NO,e}\left(t\right)+B_{BOD5}\;\cdot \;{BOD}_{e}\left(t\right) \end{array}\right)Q_{e}\left(t\right)\cdot dt,$$

where $Q_{e}$(t) represents the total effluent flow at time t, and $t_{obs}=$364 days represents the evaluation window [13].

A higher EQI value corresponds to a lower effluent quality, which indicates a higher pollutant load after passing through the treatment system. During the simulations performed by BSM2, the EQI is updated at each time step and serves as an important measure in comparing operational, control, or optimization strategies applied to the WWTP [14,15].

Given its importance in this field, it becomes a valuable target in various applications developed with NNs, since it is influenced by dynamic changes, and its prediction could help in improving the plant.

### 2.2. Simulation Scenarios

The BSM2 framework was chosen for the evaluation of the soft sensor performance because it is a standard in the scientific community for testing control and optimization algorithms. BSM2 extends previous versions and allows for a realistic evaluation of factors such as energy consumption, sludge production, and effluent quality [11].

The ability of BSM2 to operate in three different modes is a key feature that reflects its complexity, with these being the following: open-loop (OL) mode, which simulates the plant behavior without any control or feedback, closed-loop (CL) mode with ideal sensors and actuators that integrate drive signals into the control loops, and an advanced CL model with realistic sensors and actuators that take into account an additional factor such as noise [16]. By providing these basic and advanced assessments, BSM2 defines a reliable WWTP environment for the scientific community. For this study, OL simulation was used so that no external control would interfere with the process. This choice allowed the ML methods to be evaluated on the raw behavior of a WWTP, in order to not hide the model’s deficiencies and to be able to give a wider application to other plants, without being limited. Thus, all data collected under the OL mode reflect the direct response of the treatment system and provide much higher accuracy of the software sensor predictions.

Another feature integrated into BSM2 is a module capable of generating meteorological disturbances. Three scenarios are implemented to investigate how meteorological changes influence the performance of the plant, over virtual periods of 14 to 30 days, sampled at regular data intervals [11,17]. The three environmental conditions are the following:

- Dry weather, which represents the baseline conditions of BSM2 with relatively constant flow and load;
- Short-duration rain events (two to four hours), which simulate moderate rainfall that constantly increases the flow and partially dilutes the pollutants;
- Storm events, which often extend over several hours and produce episodes of intense rainfall, exceeding the usual capacity of the treatment plant and causing major fluctuations, especially observed in the case of certain parameters.

Under these conditions, it was possible to build diverse and robust datasets for training and validating the ML networks, as well as using a comprehensive mean of evaluating the proposed software sensors under both nominal and transient conditions. This ensured that the proposed models were thoroughly validated in environments that mirrored real-world variations.

### 2.3. Data Preparation

#### 2.3.1. Data Acquisition

Any complete dataset obtained from BSM2 provides dynamic results for 609 days, but not all of them can be taken into account for a formal assessment [18]. The first 245 days are defined and intended as necessary for the initialization of the process, with the remaining 364 days being used for the analysis, thus having a correct representation of the process over a whole year [19].

For this study, the five relevant compounds as part of the EQI calculation were selected and sampled at 15 min intervals, resulting in 34,944 data points for each. The input data included influent pollutant concentrations (COD, SNKj, SNO, TSS, BOD5), operational parameters (flow rate, time), and meteorological conditions (weather type). Additionally, to capture temporal dependencies, delayed values of effluent parameters, shifted by one step, were included in the model input. The output variables consisted of the corresponding effluent quality indicators (COD, SNKj, SNO, TSS, BOD5), which were used to train and evaluate the NNs. The acquisition of these data was based on both influent (such as influent and key concentrations of various pollutants) and effluent (nitrogen species, organic load, and other quality indicators) variables, including the time and weather conditions necessary to train the NNs. Given the large dataset and all the parameters included, we were able to analyze the EQI with data impacted by operational and weather conditions. In training NNs, it is important to divide the dataset to prevent overfitting of the model and to later validate their predictive performance on untrained data [20]. The structure that ensured the integrity of the test set imposed a data split into 70% for training, 15% for validation, and a remaining 15% used afterwards for testing the predictions in BSM2. Consequently, all BSM2 results reported in this study are based exclusively on the performance of the networks on the test set, which demonstrates the ability of the training to generalize to new data, instead of memorizing training examples.

Before importing the data into the NNs architectures, they required general preprocessing techniques, described in the following section, which, once applied to the data, ensured consistency in training across all networks.

#### 2.3.2. Data Preprocessing

Before training any network, preparatory steps are necessary to ensure the consistency and reliability of the data. The preparation of the data extracted from BSM2 involved cleaning the dataset to remove outliers and verify data integrity. Outliers were detected using the Z-score method with a threshold of ±3 standard deviations, identifying 482 outliers out of 174,720 data points (0.28%), which is within the expected range for a normally distributed dataset. Since this proportion aligns with statistical expectations, it confirms that the dataset does not contain an abnormal number of extreme values. Additionally, no missing data were found, ensuring the completeness of the time series.

The second procedure consisted of normalizing the data with a Min–Max Scaler. This represents mapping all variables onto a zero–one scale because the numerical ranges of the input parameters have different numerical values [21]. Regardless of the trained NN, LSTM, GRU, or Transformer, the goal was to introduce no differentiation in the data preprocessing, so that comparisons between their performances would reflect the differences in the structure of the models, rather than in data manipulation.

The third step of preprocessing was temporal segmentation, which captures time-dependent relationships and, in our integration, also applies a sliding window protocol [22]. In BSM2, this protocol specifically represents capturing 10 h of input data (40 timesteps) to predict effluent parameters 4 h ahead. According to the literature, this corresponds to the total retention time in the WWTP as modeled in BSM2. This early prediction method is an established approach in artificial neural network (ANN) strategies within BSM2 and provides sufficient time for detecting weather variations in advance [23].

Given that this study focuses on evaluation under various weather conditions such as rain and storms, the last step introduced in preprocessing was the temporal alignment of the data streams [24], ensuring that all parameters were synchronized at each sampling interval (15 min) with the meteorological disturbances generated by BSM2.

All the steps presented aimed to ensure that standardized and complete data fit each NN, so that the comparison between networks would focus on the learning model and prediction results, rather than on data customization.

### 2.4. Machine Learning Models

**Figure 1:**
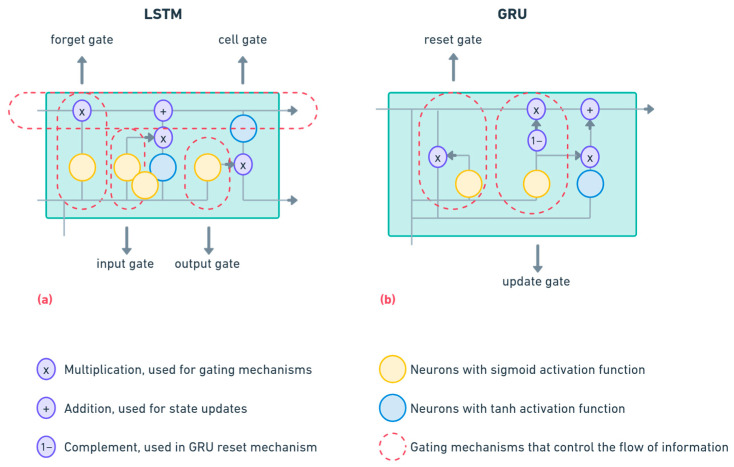
LSTM and GRU cell architectures with gate mechanisms for temporal data processing: (**a**) LSTM cell architecture; (**b**) GRU cell architecture.

In developing robust soft sensors to predict the main compounds underlying the EQI calculation, three ANN architectures were investigated. Figure 1 provides a schematic representation of LSTM and GRU cell architecture and highlights the way in which they process inputs in temporal data analysis.

#### 2.4.1. Long Short-Term Memory (LSTM) Network

LSTMs are an advanced form of recurrent neural network (RNN) that uses memory cells containing gates to regulate the flow of information over extended time intervals, as shown in Figure 1a [25]. The input gate controls the level of storage in the cell state of the new input, the forget gate determines how much of the existing state of the cell should be retained, and the output gate controls which information passes to the next layer or directly to the output [26].

Through these mechanisms, the network learns quickly from historical data and memorizes multiple time steps, which is of great importance for modeling complex, non-stationary processes, such as the dynamics of the influent and effluent of a WWTP [27,28].

We developed the LSTM network using Python 3.8 and libraries such as TensorFlow 2.7.0, scikit-learn 1.3.2, and NumPy 1.24.4 [29], and we configured a two-layer architecture with 64 neurons in the first layer and 16 neurons in the second layer, using the tanh activation function. Training was for 100 epochs with a batch size of 64, using the Adam optimizer from TensorFlow [30] and the mean squared error (MSE) loss function [31]. To increase model stability and avoid overfitting, we implemented early stopping with 12 epochs of wait without changes and a learning rate reduction mechanism when the validation loss stopped improving.

The selection of hyperparameters was performed using a Grid Search approach during the training of the LSTM network. The optimal configuration found through this process was subsequently applied to the GRU and Transformer models, ensuring a fair comparison between architectures by maintaining consistent training conditions. This approach allowed us to evaluate model performance under the same set of hyperparameters, isolating the impact of network structure rather than parameter variations.

#### 2.4.2. Gated Recurrent Units (GRU) Network

A simplified alternative to LSTM networks is represented by GRU, which is also illustrated in Figure 1b [32]. Within them, the explicit path of the cell is eliminated, using only the state and hidden cell, combining everything into a single vector and using 2 gates: the update gate, which controls how much of the hidden state is retained, and the reset gate, which takes the states as new inputs and passes them to the assumed state [33]. The advantage is that the computational load and training time are reduced but without losing the ability to capture dependencies in time series data.

The architecture and hyperparameters of the GRUs used in this paper were intentionally selected to be the same LSTM configuration to allow for comparison of their recurrent performance.

#### 2.4.3. Transformer Network

Although they were initially introduced in natural language processing (NLP) [34], Transformer architectures have adapted over time and have proven to be effective in modeling dependencies between data, even if they do not use recurrence.

**Figure 2:**
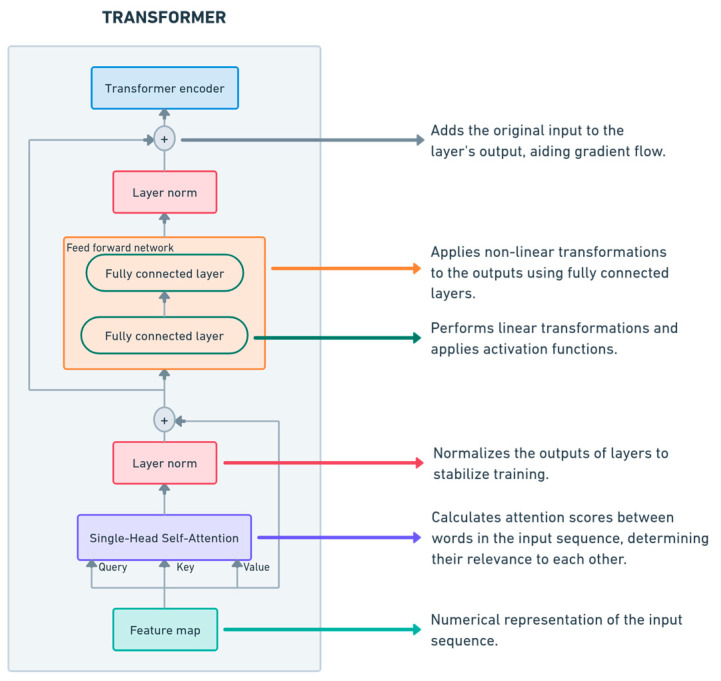
Transformer architecture highlighting single-head self-attention and feed-forward layers for temporal data processing.

A Transformer block, as shown in Figure 2, consists of a single head that allows the model to learn relationships between different time steps and feed-forward layers, which are networks that are focused on the position required to transform the attention outputs, replacing the recurrence in other types of ANNs [35].

To capture sequential dependencies between data, we implemented a Transformer encoder-based approach [35]. This allowed for a direct comparison of the two recurrent architectures (LSTM and GRU) with this feedforward-based network. The Transformer architecture consisted of two attention blocks, each with an embedding size of 64 and a feedforward layer size of 256 neurons. The network was trained in 100 epochs with a batch size of 64. The optimizer used was Adam, and the loss function used was MSE, while mean absolute error (MAE) [31] was used as the primary evaluation measure. As with the recurrent networks, to increase stability we implemented early stopping with the same patience of 12 epochs and a learning rate adaptation mechanism. The Transformer model followed the same input structure and preprocessing pipeline as the other NNs, ensuring fair comparison in terms of performance and computational efficiency.

#### 2.4.4. Meta-Classification Using Random Forest

The RF meta-classifier was developed to address the goal of this paper, which is to select the optimal NN model from LSTM, GRU and Transformer for different operational scenarios in WWTPs [36]. Unlike the NN that directly predicts effluent quality parameters, the RF classifier acts as a decision layer and analyzes the results of these models along with contextual features, such as weather conditions and temporal dynamics, to determine the most appropriate model for the current scenario.

The architecture of RF was configured with 200 decision trees, unlimited maximum depth, a minimum of two samples per leaf, and a minimum of two samples for splitting. Class weights were set to be balanced to address the non-uniform distribution of weather scenarios. These configurations were chosen to provide flexibility and accuracy in adapting to different input conditions [37].

The training dataset included the predictions for EQI parameters from LSTM, GRU, and Transformer models, contextual features such as weather types (dry, rain, storm), and temporal cues. The data were split into 80% for training and 20% for testing, allowing RF to learn models that align specific weather conditions with the most efficient NN model.

### 2.5. Evaluation Metrics

To evaluate the performance of both the ML models and the RF meta-classifier, seven evaluation metrics were selected [31]: MAE, MSE, mean absolute percentage error (MAPE), coefficient of determination (R^2^ score), dynamic time warping (DTW), a confusion matrix, and a normalized scoring system [38]. These metrics, summarized in Table 1, were able to provide a complete assessment of both the absolute magnitude of the errors and the reliability of the networks.

## 3. Results

### 3.1. Training Performance of Neural Networks

#### 3.1.1. Training Loss Evaluation

**Figure 3:**
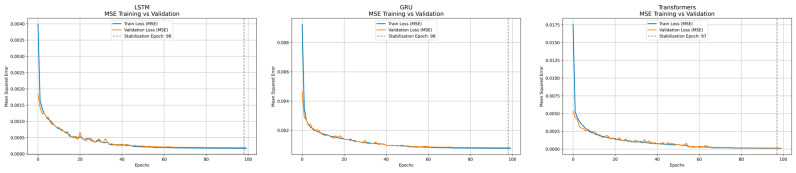
Training and validation loss across 100 epochs for LSTM, GRU, and Transformer models.

The training for each NN was performed for up to 100 epochs. A mechanism to indicate the point of stabilization of the network between epochs is displayed in each network figure with a dashed vertical line. Figure 3 illustrates the evolution of the MSE loss on both the training and validation sets, on the best validation process among the five folds’ cross-validation models.

Both LSTM and GRU show smooth convergence, with GRU reaching the lower loss values slightly earlier than LSTM. This may be due to its much simpler structure than LSTM. In contrast, Transformer requires more epochs to stabilize and reflects the complexity it has in the structure of its attention mechanism. However, Transformer reaches a lower final MSE and convergence in training. This balance between early convergence (in recurrent networks) and higher final accuracy (in attention-based model) is a factor that practitioners need to consider when selecting an architecture for effluent quality prediction.

#### 3.1.2. General Performance Metrics

In assessing the accuracy of the networks, we monitored MAE; MSE; MAPE; and R^2^ on both the training and validation sets. Table 2 shows a general presentation of these results.

From these results, it can be observed that the network trained with Transformer reflects significantly lower values of MAE and MSE and a higher R^2^ score, which suggests that it will achieve the best performance. Although LSTM and GRU obtain slightly higher error scores, both models could present strong predictive accuracy, but the decisive factor will be after their implementation in WWTP.

#### 3.1.3. Computational Requirements

Besides accuracy, an important aspect of real-world plant integration is computational cost. Table 3 summarizes important computational values, such as training time, inference time, GPU memory usage, and total number of parameters for each architecture.

These results highlight the following trade-offs:

- Training time: although GRU and Transformer have similar training times, LSTM completes fewer epochs faster, possibly due to differences in parameter initialization or smaller effective batch sizes;
- Inference speed: Transformer demonstrates the fastest inference time, suggesting that its self-attention mechanism benefits from parallelization, despite its larger number of parameters;
- Resource footprint: LSTM and GRU models use comparable GPU memory, while Transformer memory usage is slightly lower in these experiments, reflecting implementation specifics and different usage patterns;
- Model complexity: The number of Transformer parameters is substantially larger than that of recurrent models, which partly explains the improved accuracy but also increases storage requirements. Taken together, the Transformer architecture provides superior accuracy and efficient inference but requires more model complexity. In contrast, LSTM and GRU networks are easier to train and implement, making them strong candidates for scenarios where resources are constrained or where modest accuracy tradeoffs are acceptable.

### 3.2. Model Performance in Dry Weather

**Figure 4:**
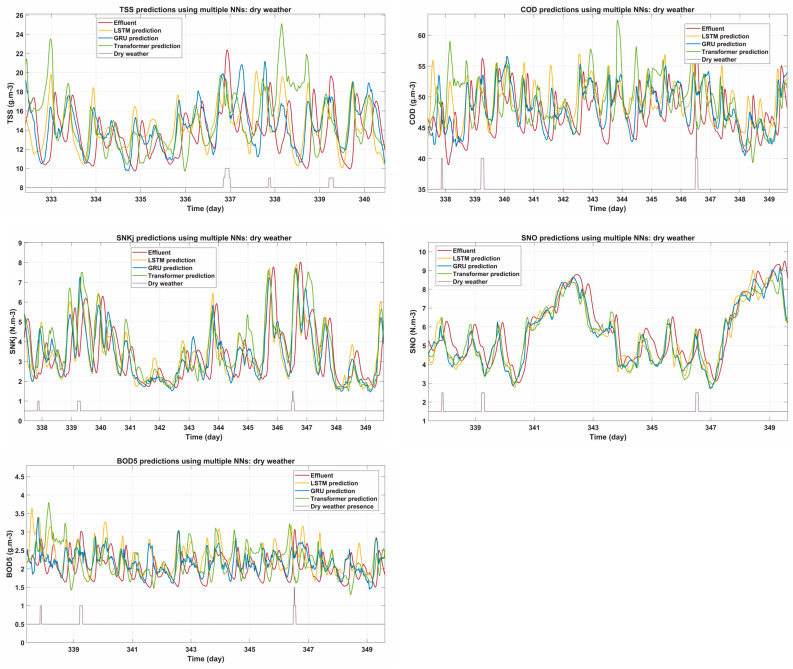
Predictions of EQI parameters (COD, SNKj, SNO, TSS, BOD5) under dry weather conditions using LSTM, GRU, and Transformer models.

Under dry weather conditions, where flow and pollutant loading remain relatively stable, the NNs showed strong predictive performance for all the parameters evaluated. Figure 4 shows the EQI parameters of effluents for different time intervals, namely COD, SNKj, SNO, TSS and BOD5, along with the predictions of LSTM, GRU and Transformer.

For COD, the Transformer model aligns best with actual effluent values, especially during daily peaks. LSTM and GRU also provide accurate predictions, although LSTM tends to slightly outperform during lower values. In the case of SNKj, GRU predictions show the most consistency, with Transformer underestimating rapid fluctuations in some cases. Similarly, for SNO, all models achieve high accuracy, although Transformer show minor delays in capturing sudden peaks.

The predictions for TSS highlight the Transformer’s ability to effectively capture larger amplitude variations, especially during high load events, while LSTM and GRU closely follow the general trend. Finally, for BOD5, LSTM and GRU demonstrate comparable performance, with only minor deviations during peaks, while Transformer predictions maintain close alignment with observed values.

Overall, the absolute prediction errors remain low for all parameters, as evident in the figure, confirming the reliability of the models under these stable conditions. The Transformer model has a slight advantage in handling high-frequency dynamics and extreme variations, but it should be noted that the above analyses are only for selected plotting intervals. Thus, the performance metrics are the ones that will make decisions based on advanced calculations.

### 3.3. Model Performance During Rainfall Episodes

**Figure 5:**
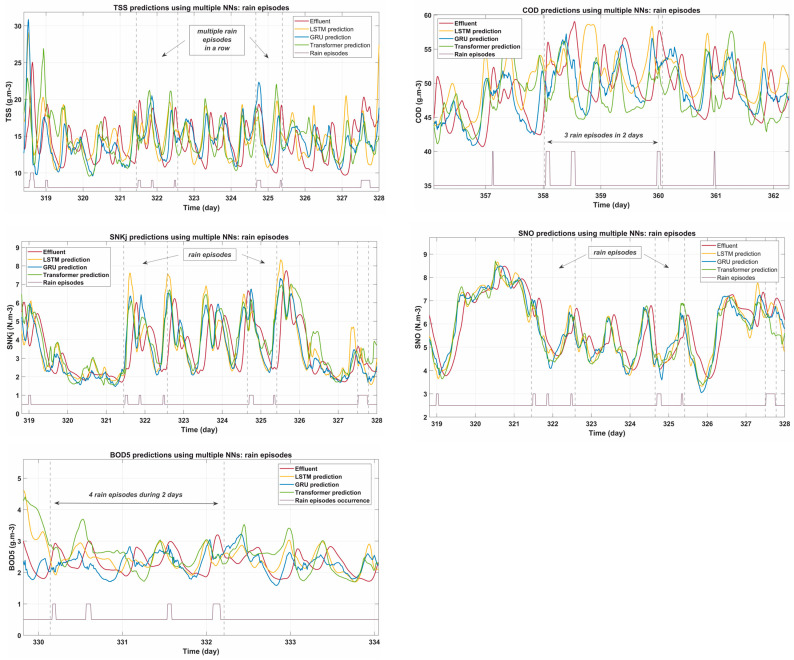
Predictions of EQI parameters (BOD5, COD, SNKj, SNO, TSS) during rainfall episodes using LSTM, GRU, and Transformer models.

Rainfall episodes significantly challenge the predictive capabilities of NN models due to sudden changes in flow rates and pollutant concentrations. Figure 5 shows the predictions generated by the three architectures during multiple rain events, illustrating their behavior under these dynamic conditions.

The predictions for BOD5 and COD reveal notable differences in how the models respond to rain-induced variability. The GRU network demonstrates robust performance, adapting quickly to sudden increases and closely following effluent trends during both the peak and stabilization phases. In contrast, the Transformer model shows strong reactivity to initial peaks but occasionally overshoots, indicating a slight delay in stabilization after rain. Although consistent in capturing overall trends, the LSTM struggled during sudden fluctuations compared to GRU and Transformer, suggesting limitations in handling rapid changes.

In the case of SNKj and SNO, the GRU and Transformer models exhibit complementary strengths. GRU provides smoother and more stable predictions, effectively capturing the overall dynamics of these parameters. Meanwhile, Transformer excels in modeling the rebound phase after rainfall, where more complex relationships between variables become critical. However, LSTM struggles in this phase, often overestimating concentrations because it is slower to adjust to post-rain stabilization.

For TSS, GRU again stands out, providing stable and accurate predictions throughout the rainfall episodes. It effectively captures both sudden increases during rainfall and subsequent stabilization trends. While Transformer also performs well, its sensitivity to rapid fluctuations is more pronounced, resulting in slightly less stability during peak periods. LSTM provides reasonable predictions, but with larger error margins, especially during peak variations, limiting its usefulness in very dynamic rainfall scenarios.

Several key trends emerge from this analysis, listed as follows:

- Initial rainfall impact: All models experience increased prediction errors at the onset of rain events. This highlights the difficulty in adapting in real time to sudden flow and pollutant peaks.
- Post-rain stabilization: GRU demonstrates the best ability to maintain stability and accuracy during the stabilization phase, while Transformer shows a slight advantage in capturing complex rebound effects.
- Overall adaptability: For all parameters, GRU consistently balances predictive responsiveness and stability, making it a strong candidate for real-time monitoring during rain episodes.

### 3.4. Model Performance During Storm Events

**Figure 6:**
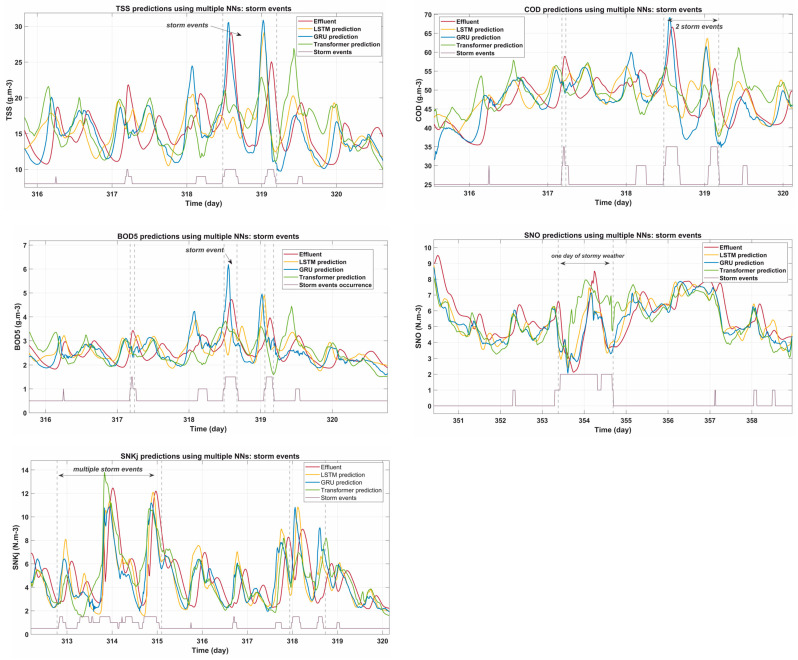
Predictions of EQI parameters (BOD5, COD, SNKj, SNO, TSS) during storm events using LSTM, GRU, and Transformer models.

Storm events pose a significant challenge to wastewater treatment systems due to sudden increases in flow and pollutant concentrations. Figure 6 illustrates the predictive performance of the models during storm episodes.

During these events, EQI parameters exhibit sharp spikes, reflecting sudden changes in environmental conditions. These increases in pollutant loadings and fluxes create complex dynamics that test the adaptability and robustness of machine learning models.

The BOD5 plot indicates that all NN models struggled to capture the largest spikes during storm events. The Transformer model, with its single-head global attention mechanism, demonstrated the fastest response to these sudden changes. However, the GRU predictions appeared slightly more consistent in tracking post-storm trends, avoiding significant over- or under-predictions.

The COD parameter, which fluctuates strongly during storms, showed the Transformer model’s superior ability to adapt to sudden changes. While LSTM encountered problems in tracking these rapid transitions, GRU provided more stable predictive behavior. All models showed higher prediction errors during the initial stages of the storm.

For SNKj, the Transformer model outperformed in capturing peak values during storm episodes, while GRU was able to maintain better accuracy during the rebound phase. The LSTM predictions, while close, tended to slightly underestimate peaks, indicating room for improvement in handling sharp variations.

The SNO parameter plots highlight the robustness of GRU in handling storm events. While Transformer excelled in detecting peaks, their predictions occasionally outperformed during the post-storm stabilization phase. LSTM predictions showed moderate accuracy but lagged during transitions. The TSS parameter showed large increases during storm events, making it particularly challenging for all models. GRU achieved the best overall balance between capturing peaks and minimizing post-storm errors. The Transformer model displayed high responsiveness but often overestimated stabilization values.

Storm events generally highlight the challenges posed by rapid and extreme variations in effluent quality. While Transformer showed superior responsiveness to peaks, GRU emerged as the most stable model for handling storm-related dynamics. Compared to LSTM, GRU demonstrated better adaptability to sudden changes, likely due to its simpler gating mechanism, which allows it to update information more efficiently in highly volatile conditions. Additionally, GRU required fewer parameters, making it computationally more efficient while still maintaining high accuracy. In this study, no major hyperparameter adjustments were made between GRU and LSTM, ensuring a fair comparison, yet GRU consistently outperformed LSTM in storm scenarios. By balancing peak detection and post-event stabilization, GRU proves to be the most suitable model for storm scenarios.

### 3.5. Comparison Across Scenarios

While the previous sections examined model performance under individual scenarios (dry weather, rain events, and storm events), this section presents an overall evaluation that combines the results of the entire test set, independent of the specific weather conditions. Rather than using an AI-based ensemble, the approach here uses only the standard error values—MAE, MSE, R^2^, and DTW—to evaluate how accurately each network predicts the individual parameters that contribute to the EQI. Table 4 reports these values, calculated on the test subset, to ensure that the results reflect the true predictive capabilities of each model, rather than a memorization of the training data.

Focusing on raw errors for TSS, COD, SNKj, BOD, and other relevant parameters, this consolidated perspective highlights the strengths and weaknesses of the LSTM, GRU, and Transformer architectures in reproducing the diverse, time-varying behaviors of the wastewater treatment plant. Furthermore, the lack of any ML-based weighting or meta-aggregation in this step ensures that the reported figures represent a mathematically transparent, data-driven view of model performance.

### 3.6. Scenario-Based Classification Using RF

The distinct weather conditions that WWTPs face, and which significantly influence the dynamics of the system, such as dry weather, moderate precipitation, and severe fog, were one of the main reasons for the development of this classifier, over the strict choice of mathematical calculations.

While in Section 3.5 we evaluated the general performance values, in this section we focus on using an RF metaclassifier to automate model selection for the three proposed scenarios. The influent flow rate served as the primary criterion for switching between scenarios, as it significantly impacts the system’s response to different weather conditions.

Since there are no predefined threshold values in the literature for model switching based on influent flow rate, a data-driven approach was applied to dynamically determine transition points. The classification of weather conditions (dry, rainy, and stormy) was based on the mean and standard deviation of influent flow fluctuations.

In this methodology, dry weather conditions were defined as periods when the influent flow remained below 1.5 times the mean flow rate, allowing for normal variations without indicating precipitation-related disturbances. Rainy conditions were identified when the flow rate exceeded this threshold but remained below the mean plus two standard deviations, capturing moderate increases due to rainfall. Finally, storm events were characterized by flow rates surpassing the mean plus two standard deviations, reflecting extreme weather conditions with sudden surges in influent volume.

By integrating these thresholds, the RF model dynamically adjusts the selected NN model based on real-time weather conditions, ensuring optimal predictive accuracy. The RF predictions are based on contextual features and historical performance data from the trained LSTM, GRU and Transformer models, as well as on the weather feature.

#### 3.6.1. Performance Metrics of RF

To assess RF’s ability to correctly classify weather conditions, precision, recall, and F1 scores were calculated using the confusion matrix for each scenario: dry (0), rain (1), and storm (2). Table 5 summarizes the classification scores, which were derived from the labeled test dataset.

The overall accuracy of the RF classifier was 98%, demonstrating its effectiveness in distinguishing between the three scenarios. The average macro F1 score (0.90) indicates a high level of robustness, although a slightly lower recall for rain (1) suggests challenges in identifying edge cases.

#### 3.6.2. RF-Based Model Classification Results

The RF metaclassifier used a normalized scoring system and contextual features, like flow variability and rainfall intensity, to dynamically assign the best-fit model for each weather condition. Table 6 summarizes the RF recommendations, along with the validation results (MAE and MSE). Table A1 from Appendix A contains the results of all NNs in each scenario.

The RF-based model selection for each weather condition is summarized in Table 7.

This classification ensures that the most suitable NN model is selected dynamically, optimizing predictive accuracy based on real-time weather conditions.

## 4. Discussions

### 4.1. Key Observations

The first thing to note is the performance of the networks across the three scenarios. GRU consistently outperformed the other models and balanced the predictive accuracy and stability across all weather conditions. Having a simpler architecture than LSTM makes it a well-suited NN for dynamic WWTP environments and offers a robust trade-off between adaptability and computational efficiency. Also worth noting here were the low computational resources it required, ranked as the second one after LSTM.

GRU demonstrated a superior ability to model sharp peaks and complicated patterns, which were especially evident during storm events, where rapid flow changes were better captured. However, the LSTM model struggled to maintain stability during the post-event stabilization phase compared to Transformer, which performed well in stable, dry weather conditions but struggled with adaptability to dynamic environmental conditions.

In terms of error trends, the occurrence of rain episodes or storm events introduced significant predictive challenges for all NNs. All models experienced more errors in the initial periods, reflecting the difficulty of adapting to sudden changes in flow rates and pollutant concentrations. Among the models, GRU showed the most reliable performance, maintaining minimal errors during post-event stabilization, a critical phase in wastewater management.

The recommendations of the RF meta-classifier highlight that GRU is much better adapted for complex and dynamic scenarios, such as rain and storms, but performs second to Transformer in stable and dry conditions. This demonstrates the potential of future hybrid approaches in performing predictions and adaptive modeling on scenarios. Thus, in the present research, GRU stands out as a versatile solution for all scenarios, and Transformer, although very capable, can benefit from additional mechanisms to establish post-event stability and to mitigate hypersensitivity during extreme variation.

### 4.2. Practical Implications and Limitations

Among the limitations we faced were the simulations based on the BSM2 framework, which, although comprehensive, cannot fully reproduce the complexity of real-world WWTP operations. BSM2 provides an idealized environment where influent conditions and operational parameters are well controlled; however, real WWTPs face additional challenges that can impact model performance. Unexpected variations in influent composition, sensor inaccuracies, unplanned system outages, and site-specific operational constraints may lead to discrepancies between model predictions and actual outcomes [39].

Additionally, models trained exclusively on BSM2 data may require recalibration when deployed in real-world facilities, as influent characteristics and treatment dynamics vary from plant to plant. Hardware and software constraints also pose potential challenges when integrating AI-driven predictive models into existing WWTP management systems. To ensure robustness and practical applicability, real-world validation on operational WWTPs is essential. Future research should focus on collecting real influent and effluent data from treatment plants, assessing model adaptability to variable conditions, and refining prediction strategies accordingly.

Furthermore, the Transformer implementation was limited to single-head attention due to MATLAB compatibility limitations [40]. While this simplified the computational process, it restricted the potential of the model, as multi-head attention could have significantly improved performance, especially under storm conditions [41]. We initially trained a multi-head attention model, which demonstrated excellent performance in capturing complex dependencies and handling extreme variations. However, due to integration constraints in Simulink, we were unable to fully implement it within the simulation framework. Consequently, we opted for the single-head attention approach to ensure compatibility while maintaining computational efficiency. The computational demand for training these models also poses challenges. The training time and resources required depend largely on the hardware used, making these approaches less accessible for facilities with limited computing capabilities [42]. Addressing these limitations will enable the adoption of AI in wastewater treatment.

### 4.3. Future Directions

As directions, we should focus on overcoming the above limitations and expanding the capabilities of AI models in wastewater management. One promising direction is to explore online learning strategies, especially for GRU networks [43]. Dynamic updates of models in response to real-time data would increase adaptability, allowing models to cope with sudden changes, such as those caused by extreme weather events [44].

Another area of development would be hybrid architectures. Combining the strengths of Transformer with LSTM or GRU layers could improve both sequential memory and attention mechanisms [45]. Bidirectional LSTM–Transformer networks and other hybrid configurations could also improve predictions by capturing more complicated temporal dependencies [46]. Integrating these hybrid models into a model predictive controller (MPC) framework, even in BSM2, would allow for dynamic optimization of WWTP operations based on real-time predictions, further enhancing efficiency and reliability [47]. Future Transformer implementations should also prioritize the integration of multi-head attention mechanisms [45]. These could allow models to process multiple temporal patterns simultaneously, improving their performance under complex conditions such as storm events.

Furthermore, implementing the models in operational WWTPs will be essential to validate their performance under real-world conditions. A structured validation approach should involve the continuous monitoring of influent and effluent parameters, comparison with model predictions, and iterative recalibration based on deviations observed in real-time data. Future studies should explore hybrid modeling approaches, combining simulated and real-world data, to enhance model generalization and improve accuracy in practical applications.

While previous studies have focused on LSTM-based models, the superior results obtained here with the GRU and Transformer architectures suggest that they are more suitable for practical implementation.

## 5. Conclusions

In WWTP, it is important to maintain effluent quality at national and international regulatory standards. When the treatment plant passes through various meteorological conditions, the EQI is directly impacted.

In this study, we aimed to evaluate three ML models, namely LSTM, GRU, and Transformer, to predict the parameters of EQI calculation in dry weather scenarios, rain episodes, and storm events. The goal was to allow for the prediction of EQI parameters, which, adjusted at the right time, can increase the effluent quality at the WWTP outlet. To generate the necessary datasets for the networks and to test their performance, we used BSM2, a complete environment for simulating a WWTP under various weather conditions.

The evaluation of the networks’ performance in all three scenarios showed that GRU adapts best to sudden variations, thus becoming the most robust model for rain and storm events. The Transformer network excelled in capturing data under dry weather conditions, while the LSTM performed reasonably well under steady-state conditions but could not handle rapid transitions.

These conclusions were reinforced by integrating a classifier built with RF, whose purpose was to automate the selection of the most suitable ML model for each type of weather. The overall classification accuracy was 98%, with RF reinforcing the results obtained by NNs.

This study introduces new AI models based on GRU and Transformer that can be integrated into WWTP applications, demonstrating the ability of soft sensors to monitor effluent quality and integrate predictions into intelligent plant management and control systems.

## Figures and Tables

**Table 1:** Evaluation metrics for ML models and RF meta-classifier [31].

Evaluation Metrics	Equation	Details
MAE	$MAE=\frac{1}{n}\sum_{i=1}^{n}\left|y_{i}-\hat{y}\right|$,	MAE measures the mean absolute deviation between predicted and actual values, and unlike MSE, MAE treats all errors equally, making it robust against large outliers. It has been particularly useful in minimizing mean deviation rather than penalizing extreme errors.
MSE	$MSE=\frac{1}{n}\sum_{i=1}^{n}{\left(y_{i}-{\hat{y}}_{i}\right)}^{2}$	MSE calculates the mean squared deviation between the predictions and the actual values, penalizing large errors more than small ones, making it effective for identifying models that minimize substantial deviations. However, its sensitivity to outliers had to be considered when evaluating the results.
MAPE	$MAPE=\frac{100\%}{n}\sum_{i=1}^{n}\left|\frac{y_{i}-\hat{y_{i}}}{y_{i}}\right|,$	MAPE expresses the prediction error as a percentage of the true value and provides a normalized measure of error, which is particularly useful when comparing models from datasets with different scales, in this case with different values for EQI parameters.
R^2^	$R^{2}=1-\frac{\sum_{i=1}^{n}{\left(y_{i}-{\hat{y}}_{i}\right)}^{2}}{\sum_{i=1}^{n}{\left(y_{i}-\bar{y}\right)}^{2}}$	The R^2^ score measures how well the model explains the variation in the actual data. A value of 1 indicates perfect predictions, and 0 means that the model is no better than using the mean. Therefore, values should be as close to 1 as possible.
DTW	$DTW(X,Y)={min}_{P}\sum_{\left(i,j\right)\in P}d(x_{i},y_{i})$	It measures the similarity between time series sequences by aligning them nonlinearly in the time dimension. It was chosen because it is suitable for sequences with similar trends but different time scales, in our case the sliding window protocol.
Confusion Matrix	$\left[\begin{array}{cc} TP & FP\\ FN & TN \end{array}\right]$	The confusion matrix was used in the classification to measure how well the RF model distinguished the weather conditions. In the formula, True Positives (TP) and True Negatives (TN) represent correct classifications, while False Positives (FP) and False Negatives (FN) indicate misclassifications.
Normalized Scoring System	$Score=\frac{MSE}{{MSE}_{max}}+\left(1-\frac{R^{2}}{R_{max}^{2}}\right)$	This system was used to rank model performance. It normalizes the MSE and R^2^ values against their maximum values for all models and ensures a balanced assessment in which both absolute error and explanation of variation are taken into account.

**Table 2:** General performance metrics for NNs on training and validation sets.

Model	MAE	MSE	MAPE	R^2^	Best K-Fold ^1^
LSTM	0.41	0.54	3.57%	0.95	2
GRU	0.46	0.74	3.99%	0.94	5
Transformer	0.006	0.00	2.34%	0.98	5

^1^ The column indicates the fold (out of 5 in the cross-validation process) that achieved the optimal performance for each NN and that was selected for further evaluation and integration into BSM2.

**Table 3:** Computational requirements for NN training.

Model	Training Time (s) ^1^	Inference Time (s)	GPU Memory Usage (MB) ^2^	Total Parameters
LSTM	226.97	0.95	68.82	25,237
GRU	293.47	1.13	70.57	19,189
Transformer	293.29	0.78	65.53	92,934

^1^ seconds; ^2^ megabytes.

**Table 4:** NN performance over the test period compared to BSM2 reference signal.

EQI Parameter	Rank	NN	MAE	MSE	DTW
TSS (mg/L)	1	GRU	2.47	11.97	16,555.11
	2	Transformer	2.89	15.37	11,207.21
	3	LSTM	3.09	16.12	19,952.33
COD (mg/L)	1	GRU	3.33	22.10	23,303.08
	2	Transformer	3.83	27.72	15,360.53
	3	LSTM	4.14	29.38	28,558.55
SNKj (mg/L)	1	Transformer	1.02	2.10	2667.36
	2	GRU	1.09	2.33	3728.43
	3	LSTM	1.14	2.61	4234.05
SNO (mg/L)	1	Transformer	0.60	0.63	2124.51
	2	GRU	0.60	0.61	2597.26
	3	LSTM	0.67	0.77	3338.26
BOD5 (mg/L)	1	GRU	0.43	1.26	3972.47
	2	Transformer	0.48	1.62	2734.55
	3	LSTM	0.51	1.45	4442.02

**Table 5:** RF performance report for classification.

Scenario	Precision	Recall	F1-Score	Support
Dry (0)	0.98	1.00	0.99	6371
Rain (1)	0.88	0.67	0.76	379
Storm (2)	0.95	0.94	0.94	239
Weighted Average	0.97	0.98	0.97	6989

**Table 6:** Best NN model performance results for each scenario using RF.

Scenario	Selected ANN	MSE	R^2^	Notes
Dry (0)	Transformer	1.96	0.97	Best for stable flow and diurnal trends.
Rain (1)	GRU	3.14	0.96	Adaptable to moderate and persistent fluctuations.
Storm (2)	GRU	5.81	0.88	Handles extreme spikes with better stability.

**Table 7:** Model selection per weather condition based on RF classification.

Weather Condition	Recommended Model	Reasoning
Dry weather	Transformer	Captures stable flow variations and diurnal patterns with high accuracy.
Rain events	GRU	Adapts well to persistent fluctuations while maintaining stable predictions.
Storms	GRU	Robust in handling extreme variations and effectively recovers after abrupt spikes.

## Data Availability

The data used in this study were generated using the Benchmark Simulation Model No. 2 (BSM2) framework. The datasets, as well as the developed software programs and code, can be made available upon reasonable request from the corresponding author.

## Abbreviations

The following abbreviations are used in this manuscript:

AI	Artificial intelligence
ANN	Artificial neural network
BOD5	Five-day biochemical oxygen demand
BSM2	Benchmark simulation model no. 2
CL	Closed loop
COD	Chemical oxygen demand
DTW	Dynamic time warping
EQI	Effluent quality index
FN	False negatives
FP	False positives
GRU	Gated Recurrent Unit
LSTM	Long Short-Term Memory
MAE	Mean absolute error
MAPE	Absolute percentage error
MATLAB	Matrix laboratory
ML	Machine learning
MPC	Model predictive controller
MSE	Mean squared error
NN	Neural network
OL	Open loop
R2	Coefficient of determination
RF	Random Forest
SNKj	Kjeldahl nitrogen
SNO	Nitrate nitrogen
TN	True Negatives
TP	True Positives
TSS	Total suspended solids
WWTP	Wastewater treatment plant

## Appendix A

**Table A1:** Complete NN model performance results for each scenario using RF.

Condition	Model	MSE	R^2^	Composite Score
Dry (0)	LSTM	2.43959	0.97068	0.20016
	GRU	2.24334	0.97341	0.18369
	Transformer	1.96981	0.97985	0.15809
Rain (1)	LSTM	3.11630	0.96360	0.28264
	GRU	3.14540	0.96599	0.28254
	Transformer	3.20165	0.96493	0.28805
Storm (2)	LSTM	6.21162	0.87506	0.26266
	GRU	5.81337	0.88798	0.24091
	Transformer	6.01183	0.86973	0.26356
